# Outcome in Caucasian patients with hepatitis B e antigen negative chronic infection: A long‐term observational cohort study

**DOI:** 10.1002/jmv.25950

**Published:** 2020-05-12

**Authors:** Özgür M. Koc, Geert Robaeys, Halit Topal, Rob Bielen, Dana Busschots, Johan Fevery, Ger H. Koek, Frederik Nevens

**Affiliations:** ^1^ Department of Gastroenterology and Hepatology Ziekenhuis Oost‐Limburg Genk Belgium; ^2^ Faculty of Medicine and Life Sciences Hasselt University Hasselt Belgium; ^3^ Department of Medical Microbiology, Medical Centre Maastricht University Maastricht The Netherlands; ^4^ School of Nutrition and Translational Research in Metabolism (NUTRIM) Maastricht University Maastricht The Netherlands; ^5^ Department of Gastroenterology and Hepatology University Hospitals KU Leuven Leuven Belgium; ^6^ Department of Abdominal Surgery University Hospitals KU Leuven Leuven Belgium; ^7^ Department of Internal Medicine, Division of Gastroenterology and Hepatology, Medical Centre Maastricht University Maastricht The Netherlands; ^8^ Department of Visceral Surgery and Transplantation University Hospital of the RWTH Aachen Germany

**Keywords:** alanine aminotransferase, Caucasian race, chronic hepatitis B, HBeAg‐negative chronic infection, HBV DNA, inactive HBV carrier

## Abstract

Sensitive polymerase chain reaction assays to measure hepatitis B virus (HBV) DNA became only available the last decade. Hence, the long‐term outcome of Caucasian patients in Western Europe with hepatitis B e antigen (HBeAg)‐negative chronic infection, especially with a baseline HBV DNA level ⩾2000 IU/mL, is still unclear. Out of a cohort of 1936 chronic HBV patients, 413 Caucasian individuals were identified with HBeAg‐negative chronic infection, defined as persistently normal alanine aminotransferase (ALT) levels and HBV DNA levels <20 000 IU/mL. During a mean follow‐up of 12 years, 366 (88.6%) maintained an HBeAg‐negative chronic infection status, whereas 25 (6.1%) developed chronic active hepatitis (CAH). In total, Nine of these 25 CAH cases were related to immunosuppression. In total, 22 (5.3%) individuals had ALT > 2 × upper limit of normal due to non‐HBV‐related causes. The cumulative probability of spontaneously developing CAH after 10 years was almost exclusively seen in patients with baseline HBV DNA level ⩾2000 IU/mL (11.7% vs 1.2%; *P* < .001). Advanced liver disease developed significantly more in patients with baseline HBV DNA level ⩾2000 IU/mL (5.2% vs 1.5%; *P* = .018) and occurred especially in patients with obesity (16.7% vs 4.2%; *P* = .049). The incidence of hepatocellular carcinoma was 0.0%. Caucasian patients with HBeAg‐negative chronic infection and baseline HBV DNA level <2000 IU/mL have an excellent long‐term prognosis in the absence of immunosuppressive therapy. However, patients with baseline HBV DNA level ⩾2000 IU/mL are at risk to develop advanced liver disease.

## Highlights

- This is the first long‐term study in Caucasian patients with HBeAg‐negative chronic infection making use of sensitive PCR assays.
- Patients with baseline HBV DNA levels >2000 IU/mL have a cumulative incidence of HBsAg loss of 10% over 10 years.
- Patients with baseline HBV DNA levels >2000 IU/mL have a favourable long‐term prognosis.
- Baseline HBV DNA level <2000 IU/mL is associated with a higher risk of advanced liver disease.

**Abbreviations**
ALT: alanine aminotransferase
anti‐HBe: antibodies against HBeAg
CAH: chronic active hepatitis
HBeAg: hepatitis B e antigen
HBsAg: hepatitis B surface antigen
HBV: hepatitis B virus
HCC: hepatocellular carcinoma
NASH: nonalcoholic steatohepatitis
PCR: polymerase chain reaction
qHBsAg: quantitative HBsAg
ULN: upper limit of normal

## 1 INTRODUCTION

Infection with hepatitis B virus (HBV) remains a global health challenge with approximately 257 million people living with chronic HBV infection, of whom 887 000 die annually from HBV‐related complications such as cirrhosis and hepatocellular carcinoma (HCC).^1^ The natural history of chronic HBV infection can be divided in four phases, taking into account the presence of hepatitis B e antigen (HBeAg), serum HBV DNA levels, and serum alanine aminotransferase (ALT) values.^2^, ^3^, ^4^

One of these phases is the HBeAg‐negative chronic infection state.^4^ This stage was previously called “inactive carrier” phase, but this terminology was abandoned since previous studies indicated that some of these patients developed advanced liver disease.^4^

According to current management guidelines on hepatitis B, therapy is not indicated in patients with HBeAg‐negative chronic infection but follow‐up for the risk of HBV reactivation, advanced liver disease, and HCC is recommended.^2^, ^3^, ^4^ Most evidence on the natural history of HBeAg‐negative chronic infection is based on studies in Asian patients, wherein the high sensitivity of polymerase chain reaction (PCR)‐based HBV DNA assays has contributed to our understanding that even low HBV DNA levels may still be associated with the risk of liver disease progression.^5^, ^6^, ^7^ However, only a limited number of studies have assessed the risk of disease progression and its predictors in Caucasian patients. Previous European studies on the natural history of patients in the chronic infection phase were limited by the short duration of follow‐up (less than 10‐year follow‐up), small groups of patients (<200 subjects), poorly defined criteria for HBeAg‐negative chronic infection, and the use of the low sensitivity branched HBV DNA assays for quantification.^8^, ^9^, ^10^, ^11^, ^12^, ^13^, ^14^, ^15^, ^16^, ^17^, ^18^

Recent sensitive PCR assays to measure HBV DNA have demonstrated that, in general, patients with HBeAg‐negative chronic infection have HBV DNA levels <2000 IU/mL. However, some patients in this phase have HBV DNA levels between 2000 and 20 000 IU/mL.^3^, ^4^ The risk of liver disease progression is unclear in this subgroup of Caucasian patients with baseline HBV DNA levels ⩾2000 IU/mL.

This is the first long‐term follow‐up study of more than 10 years in HBeAg‐negative Caucasian patients making use of the high sensitive PCR‐based assays. The main objectives were to investigate the disease outcome of those with HBV DNA levels ⩾2000 IU/mL and to find out whether a cut‐off value of serum HBV DNA level of 2000 IU/mL can predict those patients who would benefit from a strict follow‐up program and those who do not require a stringent monitoring.

## 2 PATIENTS AND METHODS

The study involved three large educational hospitals, two in Belgium and one in the Netherlands. The data were to be collected according to a protocol that followed the strengthening the reporting of observational studies in epidemiology statement with clear definitions of the study population, follow‐up, and outcome data.

### 2.1 Study population

The study identified all consecutive chronic HBV patients of the participating centers between 1 January 1987 and 31 July 2018 who fulfilled the following inclusion criteria: (a) Caucasian race, (b) persistence of HBsAg for at least 1 year, (c) the presence of antibodies to HBeAg (anti‐HBe) without HBeAg, (d) low HBV DNA (<20 000 IU/mL), (e) persistently normal ALT levels, defined as ⩾3 ALT determinations (⩽40 IU/L) at least 2 months apart over a period of at least 12 months,^5^, ^6^, ^19^ and (f) follow‐up at the enrolling center for a minimum period of 12 months. Patients previously treated with HBV antiviral agents were excluded as well as HBsAg‐positive patients with prophylactic administration of antiviral therapy. Other exclusion criteria were: (a) signs of significant fibrosis (F2 based on transient elastography values >9.0 kPa or liver biopsy), (b) patients with hepatitis D or HIV co‐infection, (c) a history of significant alcohol consumption based on a threshold of 14 units per week in men and 7 units per week in women,^20^ and (d) evidence of coexisting liver disease (eg, nonalcoholic steatohepatitis [NASH], autoimmune hepatitis).

### 2.2 Follow‐up

The observation period was calculated from the date of presentation until death or the last visit at the outpatient clinic. At the first visit in the outpatient clinic, a complete history was taken, a physical examination was conducted, and liver disease activity and severity were assessed including markers of HBV infection (eg, HBeAg and anti‐HBe, HBV DNA).^4^ Biochemical parameters and an abdominal ultrasound were recommended in all patients. Patients were monitored with periodical determinations of serum ALT and HBV DNA levels as well as for liver stiffness by transient elastography from 2006 onwards. Liver biopsy was not routinely advised and only performed in case of suspected advanced liver disease (based on laboratory results, abdominal ultrasound, and/or transient elastography) and in the suspicion of a concomitant liver disease.^4^ Results from transient elastography and liver biopsies were evaluated according to the METAVIR classification.^21^

### 2.3 Definition of clinical events

The primary outcome was the development of chronic active hepatitis (CAH), defined by increased ALT levels to more than twice the upper limit of normal (ULN) on two occasions at least 2 weeks apart with HBV DNA levels ⩾2000 IU/mL whether or not with HBeAg reversion.^2^, ^3^, ^4^ Increased ALT levels > 2 x ULN that could not be classified as CAH were designated as non‐HBV‐related cause.^22^

According to the report of the Baveno VI Consensus Workshop, advanced liver disease was suspected on transient elastography values >10 kPa.^23^ The development of cirrhosis was defined as a clinical syndrome consisting of either histological confirmation of cirrhosis or ultrasonographic findings of cirrhosis.^14^ Cirrhosis was further classified according to the Child‐Pugh score.^24^ HCC diagnosis was based on noninvasive criteria (positive lesion detected by at least two different imaging techniques) or pathology.^14^ In addition, this study evaluated the incidence of mortality and liver‐related mortality.

### 2.4 Immunosuppressive treatment

This study also assessed whether patients who developed a CAH were exposed to immunosuppressive or cancer chemotherapy. The immunosuppressive therapies were classified into those with low (eg, azathioprine), moderate (cyclosporine), and high (eg, rituximab, high‐dose corticosteroids, infliximab) risk of reactivation as previously outlined by Loomba and Liang.^25^ CAH resulting from immunosuppressive therapy was considered up to 6 months after cessation of immunosuppression, and in the case of B‐cell depleting drugs (eg, rituximab) as late as 12 months posttreatment.^26^

### 2.5 Laboratory procedures

ALT and viral markers (HBsAg, antibodies to HBsAg [anti‐HBs], HBeAg, anti‐HBe, hepatitis C and D virus antibodies, anti‐HIV) were determined using conventional serological assays. Serum samples at baseline were stored at −20°C. Up to 2002, serum HBV DNA levels were analysed on stored serum samples with the branched DNA signal amplification assay (Chiron Diagnostics, Emeryville, CA) lower limit of detection of 0.7 mEq/mL (7.00 × 10^5^ IU/mL). Thereafter, HBV DNA quantification was performed by ABI Prism 7900HT (Applied Biosystems, Thermo Fisher Scientific, Waltham, MA) with a detection limit of 100 IU/mL and from 2015 on by a commercial PCR assay (Abbott RealTime HBV assay; Abbott Molecular Inc, Des Plaines, IL) with a sensitivity of 10 IU/mL. Serum qHBsAg was measured on the Elecsys HBsAg II quant (Roche Diagnostics, Penzberg, Germany) or Architect HBsAg QT (Abbott Diagnostics, IL) assay.

### 2.6 Statistical analysis

Statistical analysis was performed with the SPSS software version 25 (IBM Corp, Armonk, NY). Continuous variables are expressed as mean  + standard deviation or medians  + interquartile range as appropriate. For the comparison of categorical variables, either the *χ* ^2^ test or the Fisher's exact test was used. The Student *t* test or Mann‐Whitney *U* nonparametric test was used to analyse continuous variables between two independent groups. The level of statistical significance was set at *P* < .05 in two‐tailed tests.

Estimates on the rate of CAH and advanced liver disease were calculated using the Kaplan‐Meier method, and the difference was determined using the logrank test. Univariate analyses (logrank tests) to identify variables associated with CAH or advanced liver disease included age at diagnosis (<40 vs ⩾40 years), sex (male vs female), obesity (yes vs no), baseline ALT level (low‐normal < 0.5 × ULN vs high‐normal 0.5 – 1 × ULN), baseline quantitative HBsAg (qHBsAg) level (<1000 vs ⩾1000 IU/mL) and baseline HBV DNA level (<2000 vs ⩾2000 IU/mL). Multivariate Cox regression analysis was not performed due to expected data sparseness, that is, CAH and advanced liver disease were not expected to occur in all converges of the abovementioned variables.

### 2.7 Ethical approval

Following the Belgian law of 7 May 2004, an approval of an ethics committee is not necessary for a retrospective non‐interventional study.

## 3 RESULTS

**Figure 1:**
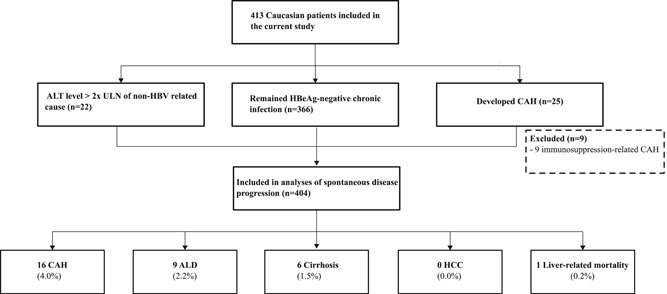
Study flowchart. ALD, advanced liver disease; ALT, alanine aminotransferase; CAH, chronic active hepatitis; HBeAg, hepatitis B e antigen; HBV, hepatitis B virus; HCC, hepatocellular carcinoma; ULN, upper limit of normal.

Out of 1936 patients with chronic HBV infection at baseline, we identified 413 Caucasians with HBeAg‐negative chronic infection according to in and exclusion criteria of this study (Figure 1). The baseline characteristics of the 413 included Caucasian patients were as follows: mean age at presentation was 34 ± 13.6 years; 210 (50.8%) were males and 81 (19.6%) had a baseline HBV DNA level ⩾2000 IU/mL. Additional information on qHBsAg level was available in 146 of 413 (35.4%) patients. In patients with detectable viremia in which genotyping could be performed: 36 of 61 (59.0%) had a genotype A and 25 of 61 (41.0%) a genotype D.

### 3.1 Chronic active hepatitis

During a mean follow‐up of 12 ± 8.3 years, 366 (88.6%) of 413 patients maintained HBeAg‐negative chronic infection, whereas 25 (6.1%) developed CAH. The remaining 22 (5.3%) individuals had ALT level > 2 × ULN of non‐HBV‐related cause (eg, nonalcoholic fatty liver disease, medication use, malignant infiltration). Of the 25 patients with CAH, 9 were considered to be the result from immunosuppressive therapy or cancer chemotherapy. Five patients received immunosuppressive therapy with a moderate risk of hepatitis B reactivation, that is, two patients were the result from post‐kidney transplant immunosuppression and three were in the setting of cancer treatment. In addition, three patients with lymphoma received rituximab containing regimens and one subject was treated with high‐dose (>20 mg orally daily) corticosteroids for rheumatic disease.

Table 1 illustrates the baseline characteristics of the 404 Caucasian patients after excluding those nine patients with CAH due to immunosuppressive therapy. Thus, the prevalence of spontaneous development to CAH in the study population was 16 of 404 (4.0%) (Figure 1).

**Table 1:** Baseline characteristics of 404 Caucasian patients with hepatitis B e antigen negative chronic infection

Characteristics	All (n = 404)	Remained HBeAg‐negative chronic infection (n = 366)	CAH (n = 16)	*P* value
Baseline age, y	34 ± 13.5	34 ± 13.3	31 ± 12.2	.511
Sex, males, %	205 (50.7)	181 (49.5)	10 (62.5)	.307
Obesity^a^, %	32 (7.9)	27 (7.4)	1 (6.3)	1.000
Baseline ALT, IU/L	24 ± 8.2	24 ± 8.2	25 ± 7.4	.427
Baseline qHBsAg level, IU/mL^b^, ^c^	365 ± 2010.7	362 ± 1613.9	1965 ± 5788,0	.525
Baseline HBV DNA, log IU/mL^b^	2.3 ± 3.11	2.3 ± 3.09	3.3 ± 0.69	.003
HBV DNA >2000 IU/mL, %	77 (19.1)	64 (17.5)	9 (56.3)	.001

*Note:* Values shown as mean ± standard deviation or as n (%).

A total of 366 individuals remained HBeAg‐negative chronic infection, 16 developed spontaneous CAH, while the remaining 22 patients had increased ALT levels > 2 × ULN of non‐HBV‐related cause.

Abbreviations: ALT, alanine aminotransferase; CAH, chronic active hepatitis B; HBeAg, hepatitis B e antigen; HBV, hepatitis B virus; qHBsAg, quantification of hepatitis B surface antigen; ULN, upper limit of normal.

^a^ Obesity was defined as BMI >30 kg/m^2^.

^b^ Mann‐Whitney U nonparametric test was used instead and median ± interquartile range were shown as appropriate.

^c^ Information on qHBsAg was available in 146 of 404 (36.1%), 130 of 366 (35.5%), and 6 of 16 (37.5%) among all 404 patients, patients who remained HBeAg‐negative chronic infection and those with spontaneous CAH development, respectively.

**Figure 2:**
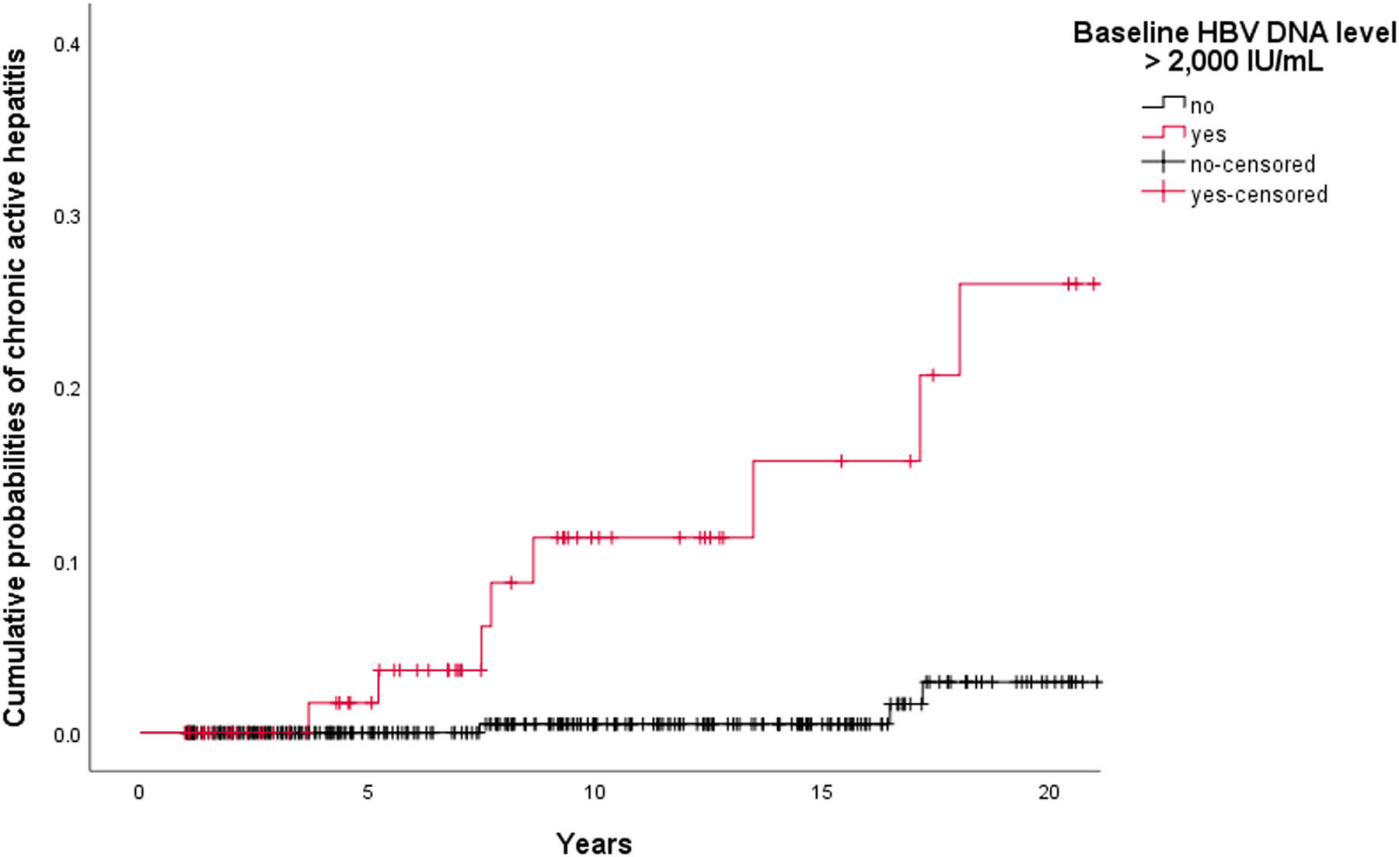
**Table:** 

The cumulative probabilities of CAH development were 1 of 283 (0.4%), 7 of 196 (3.6%), and 8 of 131 (6.1%) at 5, 10, and 15 years follow‐up, respectively.In univariate analyses (logrank tests) among the 404 Caucasian patients, baseline HBV DNA level ⩾2000IU/mL (11.7% vs 1.2%; *P* < .001) and qHBsAg level ⩾1000IU/mL (7.1% vs 1.0%; *P* = .015) were the only factors significantly associated with the occurrence of CAH. Figure 2 shows the cumulative probabilities of spontaneous CAH development in two groups, that is, baseline HBV DNA levels <2000IU/mL and those with baseline HBV DNA levels ⩾2000IU/mL. Among patients with baseline HBV DNA level ⩾2000IU/mL (n = 77), qHBsAg level ⩾1000IU/mL identified patients with a higher risk to develop CAH (*P* = .002): it was 28.6% for patients with a qHBsAg level ⩾1 000IU/mL and 0.0% if qHBsAg level <1000IU/mL.

**Time, y**	0	1	2	3	5	10	15	20
**Baseline HBV DNA level** >**2000 IU/mL**	77	75	65	59	52	27	18	13
**CAH development**	…	0	0	0	1	4	1	2
**Censored**	…	2	10	6	6	21	8	3
**Time, y**	0	1	2	3	5	10	15	20
**Baseline HBV DNA level** <**2000 IU/mL**	327	326	294	272	229	161	104	57
**CAH development**	…	0	0	0	0	1	0	2
**Censored**	…	1	32	22	43	67	57	45 Cumulative probabilities of spontaneous chronic active hepatitis development by baseline hepatitis B virus DNA level (n = 404). A total of 404 Caucasian patients with HBeAg‐negative chronic infection were analysed after excluding nine individuals with immunosuppression‐related CAH occurrence. Among this group, progression to CAH was higher in patients with baseline HBV DNA level ⩾2000 IU/mL than in patients with baseline HBV DNA level <2000 IU/mL (*P* < .001, logrank test). Cumulative probabilities of CAH were 1 of 53 (1.9%) vs 0 of 229 (0.0%), 5 of 32 (15.6%) vs 1 of 162 (0.6%), and 6 of 24 (25.0%) vs 1 of 105 (1.0%) at 5, 10, and 15 years follow‐up, respectively. ^†^Patients were censored on the date of last outpatient clinic visit. HBeAg, hepatitis B e antigen; CAH, chronic active hepatitis

### 3.2 Follow‐up of serum HBV DNA levels and HBsAg

Longitudinal follow‐up of serum HBV DNA levels revealed that HBV DNA levels exceeded 2000 IU/mL in 151 of 404 (37.4%) Caucasian patients during follow‐up vs 77 of 404 (19.1%) at baseline and that 35 of 404 (8.7%) patients developed an HBV DNA level ⩾20 000 IU/ml. Spontaneous HBsAg loss occurred in 0.94 per 100 persons‐years. The cumulative incidences of HBsAg loss were 9 of 291 (3.1%), 19 of 206 (9.2%), and 32 of 145 (22.1%) at 5, 10, and 15 years follow‐up, respectively. HBsAg loss was 11.5% in the group with HBV DNA level <2000 IU/mL and 4.9% in those with HBV DNA >2000 IU/mL, *P* = .080. In the group of patients with HBsAg loss (n = 42), anti‐HBs appeared in 29 (69.0%) patients. The cumulative appearances of anti‐HBs for 1, 2, 5, and 10 years were 5.1% (2 of 39), 13.2% (5 of 38), 40.0% (14 of 35), and 59.4% (19 of 32).

### 3.3 Clinical outcome

A total of 73 (18.1%) out of the 404 Caucasian patients underwent percutaneous liver biopsy during follow‐up on suspicion of progressive liver disease. Histologically proven advanced liver disease was present in 5 of 73 (6.8%) patients. Overall, the cumulative probabilities of advanced liver disease were 0 of 285 (0.0%), 2 of 195 (1.0%), and 4 of 127 (3.1%) at 5, 10, and 15 years of follow‐up, respectively.

**Figure 3:**
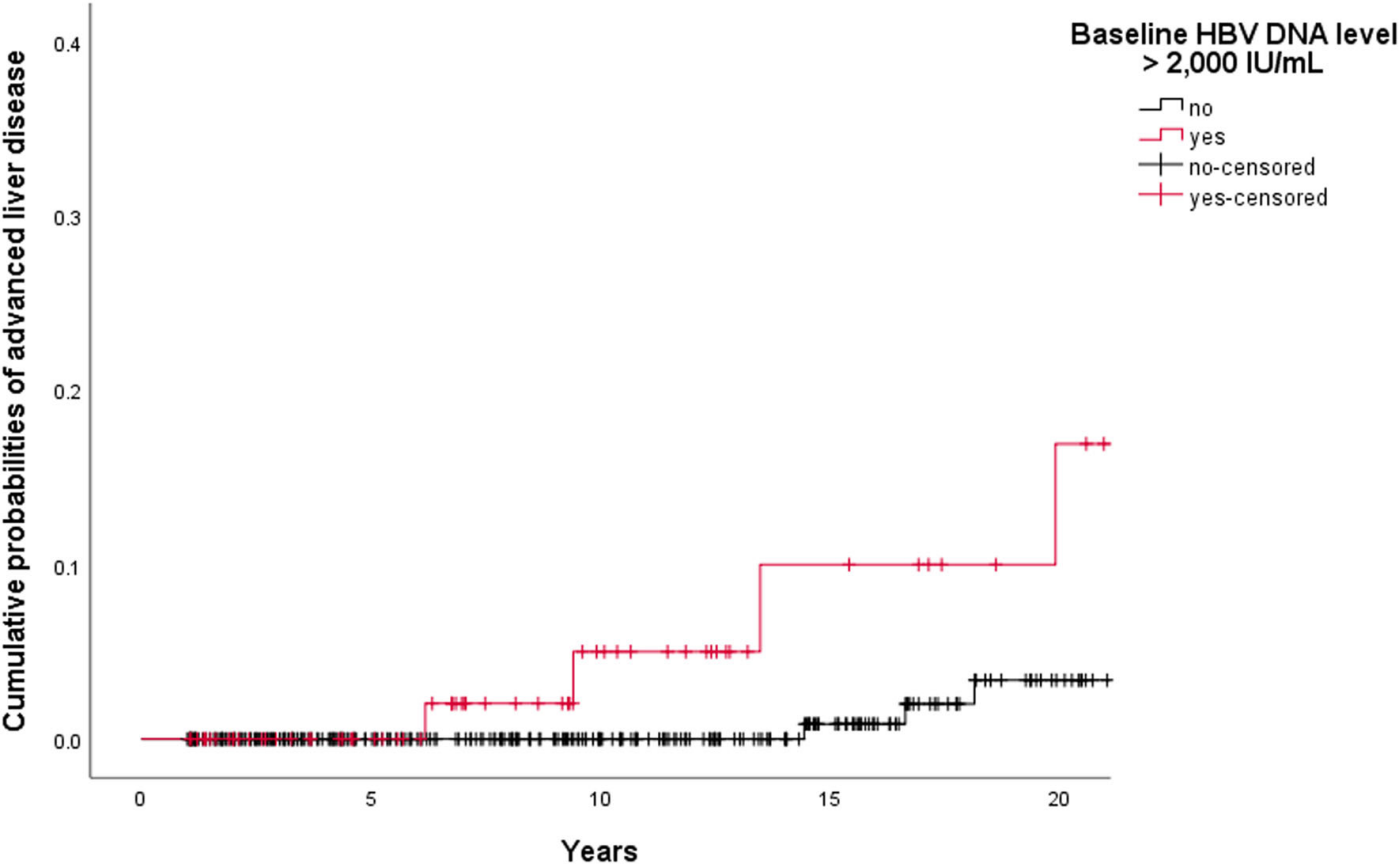
**Table:** 

Risk predictors for advanced liver disease in the univariate analyses (logrank tests) among the 404 patients were male sex (3.9% vs 0.5%; *P* = 0.029; File S1) and baseline HBV DNA level ⩾2000 IU/mL (5.2% vs 1.5%; *P* = .018). Among patients with baseline HBV DNA level ⩾2000 IU/mL (n = 77), obesity was associated with a higher risk of advanced liver disease (16.7% vs 4.2%; *P* = .049). In the group of patients with baseline HBV DNA level <2000 IU/mL (n = 327), none of the variables were significantly associated with a lower risk of progression to advanced liver disease. Figure 3 illustrates the progression to advanced liver disease in patients with baseline HBV DNA level <2000 IU/mL against those with baseline HBV DNA levels ⩾2000 IU/mL.

**Time, y**	0	1	2	3	5	10	15	20
**Baseline HBV DNA level** >**2000 IU/mL**	77	76	66	60	53	29	17	11
**Advanced liver disease**	**…**	0	0	0	0	2	1	1
**Censored**	…	1	10	6	7	22	11	5
**Time, y**	0	1	2	3	5	10	15	20
**Baseline HBV DNA level** <**2000 IU/mL**	327	326	296	274	231	163	105	58
**Advanced liver disease**	…	0	0	0	0	0	1	2
**Censored**	…	1	30	22	42	68	57	45 Cumulative probabilities of progression to advanced liver disease by baseline hepatitis B virus DNA level (n = 404). After excluding nine individuals with HBV reactivation due to immunosuppressive therapy, progression to advanced liver disease was higher in patients with baseline HBV DNA level >2000 IU/mL than in patients with baseline HBV DNA level <2000 IU/mL (*P* = .018, logrank test). Cumulative probabilities of advanced liver disease were 0 of 53 (0.0%) vs 0 of 231 (0.0%), 2 of 31 (6.5%) vs 0 of 163 (0.0%), and 3 of 20 (15.0%) vs 1 of 106 (0.9%) at 5, 10, and 15 years follow‐up, respectively. HBV, hepatitis B virus. ^†^Patients were censored on the date of last outpatient clinic visit

In total, 6 of 404 patients (1.5%) developed cirrhosis. Five had Child‐Pugh score A and one died due to acute‐on‐chronic liver failure. There were no reports of HCC. The mean time to cirrhosis development was 17 ± 4.5 years.

A total of 381 of 404 (94.3%) were alive at the end of follow‐up. Death occurred in 23 (5.7%) patients and among 4 of them reason of death was unknown. In the remaining 19 patients, there was 1 liver‐related mortality reported. Malignant neoplasms were the leading cause of death, accounting for nine cases of death. Chronic obstructive pulmonary disease, myocardial infarction, pneumonia, end‐stage renal disease, and enteric fever explained the remaining 10 deaths.

## 4 DISCUSSION

The term “inactive carrier” for patients with HBeAg‐negative chronic infection was abandoned as Asian studies demonstrated that even low HBV DNA levels may still be associated with the risk of liver disease progression.^5^, ^6^, ^7^ The introduction of sensitive PCR‐based HBV DNA assays over the last decade has demonstrated that most patients with HBeAg‐negative chronic infection have HBV DNA levels <2000 IU/mL. However, some patients in this phase might have HBV DNA levels between 2000 and 20 000 IU/mL.^3^, ^4^ Considering the fact that sensitive PCR to measure HBV DNA levels only became available the last decade, this is the first study to assess the 10 years or more disease outcome of Caucasian patients in Western Europe with HBeAg‐negative chronic infection based on stringent criteria and the use of sensitive PCR‐based HBV DNA assays.

Only few other studies in the past have addressed the follow‐up of HBeAg‐negative Caucasian patients with persistently normal ALT levels and HBV DNA levels <20 000 IU/mL based on sensitive PCR assay.^8^, ^10^, ^11^, ^16^, ^17^ Limited by a short follow‐up time varying from 1 to 5 years, none had developed cirrhosis and no HCC or liver‐related mortality was reported in these studies. These studies were also hampered by the small number of study participants (n = 35‐195).

In studies with a longer follow‐up, the prevalence of cirrhosis varied widely from 0.4% to 17.5%.^12^, ^13^, ^14^, ^15^ Correlation between baseline HBV DNA level and worse prognosis was not evaluated in these studies.^12^, ^13^, ^14^, ^15^ Moreover, in the prior studies, patients’ serum HBV DNA level was quantified by an insensitive hybridization method. Hence, some patients may have been misclassified as HBeAg‐negative chronic infection without use of PCR‐based assays.

That the level of viremia can be important has been illustrated in the REVEAL study in Asian patients. There was an increased risk of cirrhosis, HCC, and liver‐related mortality in HBeAg‐negative patients with baseline normal ALT level and HBV DNA level ⩾2000 IU/mL.^7^

There are only a few studies that have addressed the issue of CAH development in Caucasian HBeAg‐negative patients with persistently normal ALT values and HBV DNA levels below 20 000 IU/mL on sensitive PCR‐based assays.^8^, ^9^, ^11^, ^17^ With a limited follow‐up between 1 and 5 years, the overall prevalence of progression to CAH in these studies was 0.0% in patients with HBV DNA level <2000 IU/mL and varied widely from 0.0% to 50.0% in those with HBV DNA level ⩾2000 IU/mL. Our finding of spontaneous progression to CAH over 10 years was 1.2% and 11.7% in those patients with baseline HBV DNA level <2000 IU/mL and HBV DNA level ⩾2000 IU/mL, respectively. This is lower than previously reported and could be ascribed to differences in study population, follow‐up duration, and the use of different criteria for CAH.^8^, ^9^, ^11^, ^17^ Moreover, the number of patients with baseline HBV DNA level ⩾2000 IU/mL in the previous studies was small (n = 4‐46).^8^, ^9^, ^11^, ^17^ In the current cohort, 81 patients had a baseline HBV DNA level ⩾2000 IU/mL. In line with our findings, Papatheodoridis et al^9^ confirmed the relation between baseline HBV DNA level ⩾2000 IU/mL and the risk of HBV reactivation in patients in Greece.

Recent data have shown that hepatitis B surface antigen quantification might be helpful in the decision on the frequency of follow‐up in such patients.^2^, ^3^, ^4^, ^27^ However, this test is not routinely available in daily practice. In line with prior studies, our patients—in which we could do this test—had a median qHBsAg level <1000 IU/mL and qHBsAg level could identify patients at higher risk of progression in the subgroup with baseline HBV DNA level ⩾2000 IU/mL.^11^, ^27^

As a consequence of the growing obesity epidemic, more and more chronic HBV patients with coexisting NASH are expected.^28^ NASH is an independent risk factor for cirrhosis and HCC, and in our study obesity was also a risk factor for advanced liver disease among those patients with baseline HBV DNA level ⩾2000 IU/mL.^28^

We could also confirm that CAH was triggered by immunosuppressive therapy or cancer chemotherapy. In that respect, we found that immunosuppression was associated with the development of CAH in more than one‐third of the patients, underlying the importance of screening for hepatitis B before starting immunosuppressive therapies.^2^, ^3^, ^4^

One limitation of the current study is that variables such as genotype, precore/core mutations, and platelets level were not included in the current study to assess their association with disease progression. Genotype and precore/core mutations are not routinely determined in standard practice. After all, this study aimed to investigate the long‐term disease outcome and their predictors among Caucasian patients with HBeAg‐negative chronic infection in real‐life daily practice.

In conclusion, Caucasian patients from Western Europe with HBeAg‐negative chronic infection and baseline HBV DNA levels <2000 IU/mL have a favorable condition without any risk of HCC and with a cumulative incidence of HBsAg loss of approximately 10%. This is in contrast to patients with baseline HBV DNA levels ⩾2000 IU/mL who are, especially in the presence of obesity, at risk for advanced liver disease.

## CONFLICT OF INTERESTS

The authors declare that there are no conflict of interests.

## Supporting information

Supporting information
