# Supplementary material for: Outcome in Caucasian patients with hepatitis B e antigen negative chronic infection: A long‐term observational cohort study
Source: J Med Virol. 2020 May 12;92(12):3373–80. doi: 10.1002/jmv.25950 (PMC7687269; doi:10.1002/jmv.25950)
Supplement: Supplementary file 1 — Supporting information [file JMV-92-3373-s001.docx]

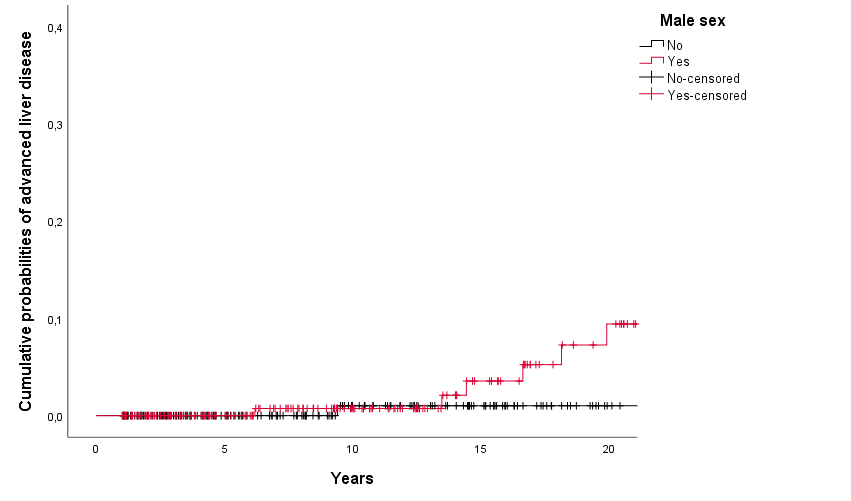


**Time (years)** 0 1 2 3 5 10 15 20

**Male sex** 205 204 183 168 145 102 62 41

**Advanced liver disease -** 0 0 0 0 1 2 3

**Censored**  - 1 21 15 23 42 38 18

**Time (years)** 0 1 2 3 5 10 15 20

**Female sex** 199 198 179 166 139 90 60 28

**Advanced liver disease -** 0 0 0 0 1 0 0

**Censored**  - 1 19 13 27 48 30 32

**Supplementary file S1. Cumulative probabilities of progression to advanced liver disease by gender (n=404).** Male sex predicted a higher risk of advanced liver disease progression (*p* = 0.029, log-rank test). Cumulative probabilities of advanced liver disease were 0/145 (0.0%) vs 0/139 (0.0%), 1/103 (1.0%) vs 1/91 (1.1%), 3/65 (4.6%) vs 1/61 (1.6%) at 5, 10 and 15 years follow-up, respectively.

^Abbreviations: HBV: hepatitis B virus.^

^†Patients were censored on the date of last outpatient clinic visit.^
